# Assessment of Surface Disinfection Effectiveness of Decontamination System COUNTERFOG® SDR-F05A+ Against Bacteriophage ɸ29

**DOI:** 10.1007/s12560-022-09526-z

**Published:** 2022-07-19

**Authors:** Cristina del Álamo, Ángela Vázquez-Calvo, Antonio Alcamí, Juan Sánchez-García-Casarrubios, José Luis Pérez-Díaz

**Affiliations:** 1 Escuela Politécnica Superior UAH, Universidad de Alcalá, Campus Universitario, Ctra. Madrid-Barcelona km 33,600, 28805 Alcalá de Henares, Spain; 2 Centro de Biología Molecular Severo Ochoa (CBMSO), Consejo Superior de Investigaciones Científicas (CSIC) and Universidad Autónoma de Madrid (UAM), Madrid, Spain; 3 San Jorge Tecnológicas S.L. (SJT), Madrid, Spain

**Keywords:** COUNTERFOG, Surface disinfection, Virus, Decontamination, Inactivation, Bacteriophage phi29

## Abstract

The experience of COVID19 pandemic has demonstrated the real concern of biological agents dispersed in the air and surfaces environments. Therefore, the need of a fast and large-scale disinfection method has arisen for prevention of contagion. COUNTERFOG® is an innovative technology developed for large-scale decontamination of air and surfaces. The objective of this study is to assess experimentally the effectiveness of COUNTERFOG® in disinfecting viral-contaminated surfaces. We also aim to measure the necessary time to disinfect said surfaces. Stainless steel surfaces were contaminated with bacteriophage φ29 and disinfected using COUNTERFOG® SDR-F05A+, which uses a sodium hypochlorite solution at different concentrations and for different exposure times. A log reduction over 6 logs of virus titer is obtained in 1 min with 1.2% sodium hypochlorite when the application is direct; while at a radial distance of 5 cm from the point of application the disinfection reaches a reduction of 5.5 logs in 8 min. In the same way, a higher dilution of the sodium hypochlorite concentration (0.7% NaOCl) requires more exposure time (16 min) to obtain the same log reduction (> 6 logs). COUNTERFOG® creates, in a short time and at a distance of 2 m from the point of application, a thin layer of disinfectant that covers the surfaces. The selection of the concentration and exposure time is critical for the efficacy of disinfection. These tests demonstrate that a concentration between 0.7- 1.2% sodium hypochlorite is enough for a fast and efficient ɸ29 phage inactivation. The fact that ɸ29 phage is more resistant to disinfection than SARS-CoV-2 sustains this disinfection procedure.

## Introduction

Currently, one of the main concerns to public health is respiratory and high transmissible diseases. Due to global pandemic originated by severe acute respiratory syndrome coronavirus 2 (SARS-CoV-2), several studies have been taken to investigate the mode of transmission of the virus and the factors that affect its infectivity. The principal strategies taken for infectious diseases besides therapeutic solution or vaccine are the control of transmission and the search of disinfection methods.

When a virus spreads through respiratory transmission, it does so either with virions suspended on large droplets (particles larger than 100 μm that fall to the ground within 6 feet) or fine aerosols (particles smaller than 100 μm that can remain suspended in the air for prolonged periods) expelled from the respiratory tract of the primary case patient (Meyerowitz et al., 2021; Prather et al., 2020). Fomites and aerosols are two main transmission channels for many pathogens. For example, the airborne transmission of SARS-CoV-2 has been extensively studied, a few studies revealed that viable SARS-CoV-2 can be present in aerosols generated by a coronavirus disease 19 (COVID-19) patient (Lednicky et al., 2020; Santarpia et al., 2020), remaining infectious in aerosols for 3 to 16 h and being able to survive at room temperature and relative humidity of 65% for a few days (Aboubakr et al., 2021).

Respiratory droplets may be expelled when infected people sneeze, cough, speak, etc. Large droplets are too heavy to remain airborne and will eventually fall, subsequently contaminating the surfaces below. Though respiratory viruses can be transmitted via contact with surfaces, in some cases (for example SARS-CoV-2) the relative contribution of fomites to transmission remains unknown (Moschovis et al., 2021). In some studies the relevance of contact transmission of COVID-19 has been questioned because viral infectivity is not recovered from contaminated surfaces (Goldman, 2020), determining no relevant risk of infection through contact with surfaces in public areas (Zedtwitz-Liebenstein, 2022). However, persistence and stability of SARS-CoV-2 has been extensively studied: up to 50% of high-touch hospital surfaces tested positive for the presence of SARS-CoV-2 RNA (Bueckert et al., 2020; Wu et al., 2020); persistence of SARS-CoV-2 is significantly low on copper, latex and less porous but is higher in other common surfaces like stainless steel, plastics, glass and highly porous fabrics (Aboubakr et al., 2021). Contamination of these surfaces demonstrates the occurrence of hand-to-surface inoculations. In any case, according to both the Center for Disease Control and the World Health Organization, contact transmission is one of the main transmission routes of infectious diseases worldwide (Kim et al., 2022). Therefore, to reduce the spread of infectious diseases and nosocomial infections, new approaches to surface sanitization are urgently required.

A limitation for the study of human viruses, as SARS-CoV-2, is the need of biosafety level 3 (BSL-3) facilities (Kaufer et al., 2020). Surrogates are often selected to model highly infectious pathogens. Bacteriophages are commonly used as surrogates for human viruses, as they are similar in terms of size, shape, morphology, surface properties, mode of replication, and environmental persistence, yet are non-infectious for humans (Gallandat & Lantagne, 2017). Safety is the major benefit of using nonpathogenic surrogate organisms, these organisms are also easily cultivate and testing is rapid and inexpensive (Gallandat & Lantagne, 2017; Sinclair et al., 2012). Bacteriophage ɸ29, previously suggested as good, safe, and easy to work with (Twomey et al., 2003), has been used in this study as a model surrogate for studying surface disinfection. ɸ29 phages contain double-stranded DNA molecule (dsDNA) and the virions have prolate icosahedral heads and are tailed. They belong to the *Podoviridae* family and they usually infect *Bacillus subtilis* and several strains of *Bacillus licheniformis* and *Bacillus pumilus* (Horcajadas & Salas, 2001). Bacteriophage ɸ29 is a small non-enveloped virus, it is one of the smallest *Bacillus subtilis* phage (Anderson et al., 1966).

The selection of an appropriate decontamination strategy is a main challenge when talking about air- dispersed agents that can be inhaled and penetrate the lungs. Widespread releases of these agents, whether they are chemical, biological, radiological or nuclear (CBRN) can be in the form of intentional dissemination (bioterror attack) or natural outbreak (Wyrzykowska-Ceradini et al., 2019). COUNTERFOG® system has been proposed as a rapid decontamination and disinfection technology of both air and surfaces, that uses dynamic submicrometric fog cones (Pérez- Díaz et al., 2021). It was designed for collapsing all kinds of dispersed agents using a fog made of a solution that can also contain any kind of neutralizing component (Pérez-Díaz et al., 2018; Sánchez García- Casarrubios et al., 2018). The application of disinfectants as fog or gaseous offers advantages over liquids, sprays and wipes in large-scale decontamination because they are easily dispersed, may penetrate in surfaces and do not require extensive training in its use (Rogers et al., 2009; Wood et al., 2013).

It has been previously demonstrated that this new technology can either be used against biological agents, being able to efficiently reduce the amount of viable of *Bacillus thurigiensis* spores from the air after 5 min of the fog release (Martín-Pérez et al., 2018); or other inert agents, COUNTERFOG® system was able to reduce the number of solid particles from the combustion of the Diesel in a percentage close to 100% in the case of particles of sizes 2.5 μm, 5 μm and 10 μm diameter in a time not exceeding 30 min (Pérez-Díaz et al., 2019). COUNTERFOG® is presented as an efficient and safe to use alternative for large scale decontamination and disinfection of submicrometric agents that suppose a threat for the environmental and public health.

The present study aims to assess the effectiveness of COUNTERFOG® technology in disinfecting viral- contaminated surfaces. In order to test this, stainless steel coupons were contaminated with bacteriophage ɸ29 and COUNTERFOG® technology was applied. Sodium hypochlorite was used as a disinfectant and applied with COUNTERFOG® system, in order to be able to evaluate the effectiveness of this disinfectant when combined with this technology. Furthermore, in this study, we evaluate the degree of disinfection in terms of radial distance of application of COUNTERFOG® technology and the exposure time necessary for an efficient disinfection.

## Methods

### Target Organism Propagation and Purification

Bacteriophage ɸ29 (kindly provided by Dionisio Ureña from CBMSO) was propagated in *Bacillus subtilis* host cells in LB medium supplemented with 10 mM MgSO_4_ and 10 mM glucose. Briefly, *Bacillus subtilis* culture (OD_420nm_ = 0.45), was infected with bacteriophage ɸ29 (MOI = 5, multiplicity of infection) and incubated at 37 °C with shaking until total lysis; clarify by centrifugation at 5000×*g* for 20 min at 4 °C. Then ɸ29 virus was precipitated with 10% of polyethylene glycol 6000 overnight at 4 °C. The precipitate was homogenized and centrifuged at 1000×*g* and 4 °C for 40 min. The pellet was washed at least three times by resuspension in 2 × phage diluent solution (50 mM TrisClH pH7.8, 10 mM MgCl_2_, 100 mM NaCl, 0.1% Tween-20) and centrifuged at 10000×*g* for 15 min in a glass tube. The supernatant was collected and keeping after each wash. Finally, the virus contained in the supernatants was concentrated using an Amicon®100 K. The virus was stored at − 20 °C in a phage diluent solution 2 × and glycerol (1:1). Viral titer was determined using the two-step double-agar overlay method (Santos et al., 2009).

### Test Material and Coupons Inoculation for Disinfection Tests

The material used for the surface disinfection testing by COUNTERFOG® equipment was stainless steel. This material is the substrate used in some disinfectant testing standards, non- porous material of common use, kitchen areas and public facilities in which viruses can persist for a few days (Riddell et al., 2020). Samples of this material were cut in coupons of 20 × 9 mm.

Prior to inoculation with bacteriophage ɸ29, stainless steel coupons were sterilized in a Pasteur oven at 160 °C. According to UNE-EN 14476:2014 + A2 and UNE-EN 16777:2019 standards, it is recommended that the minimum titer of the virus suspension be 10^8^ TCID_50_/ml, it must be high enough to be able to observe the logarithmic reduction (AENOR, 2019, 2020). Each test coupon was laid flat and contaminated with 10 µl of a viral dilution containing ~ 2 × 10^8^ plaque forming units (PFU). The suspension was transferred and spread over the entire surface of each test coupon using the pipette tip. The coupons were dried for 1–2 h at room temperature.

### Surface Disinfection Procedure Using COUNTERFOG® System

A description of COUNTERFOG® can be found in Pérez-Díaz et al. (2018). This system is based in a nozzle able to provide a large amount of fog, which was engineered to work requiring only compressed air and water supply. The principle of COUNTERFOG® is to provide a fog, mainly made of water droplets sized between 2.5 and 20 µm. This fog will interact with the dispersed agent providing chances for collapsing and neutralization (Pérez-Díaz et al., 2018). Suspended particles collapse with the fog nano-sized liquid droplets and are dragged by the cone, posing on the ground or onto surfaces (Pérez-Díaz et al., 2021).

Biological agents can be rapidly eliminated when the fog cones are projected with a disinfectant onto surfaces by covering them with a thin layer of biocide (a few microns thick) (Pérez-Díaz et al., 2021).

For this study we used the COUNTERFOG SDR-F05A+ device and for all the decontamination runs the nozzle was set to its maximum opening. The fogs were made of air and different concentrations of commercial bleach (Bosque Verde) (sodium hypochlorite concentration ≈ 3.6–4%).

**Fig. 1:**
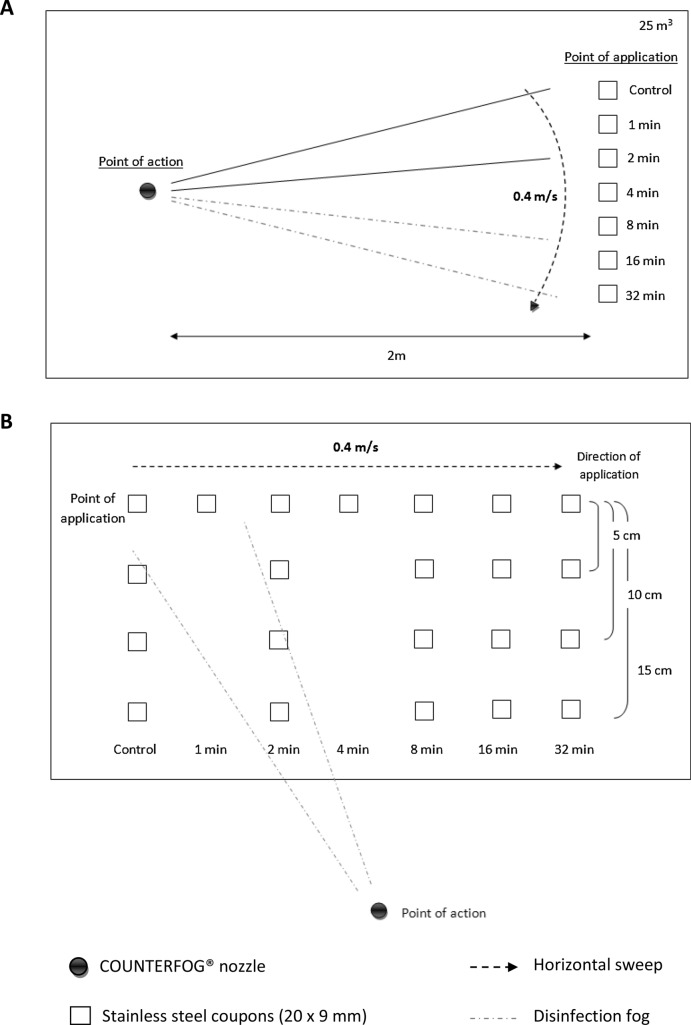
Schematic diagram of surface disinfection procedure using COUNTERFOG SDR-F05A+. **A** Top view of the position of stainless steel coupons in the disinfection assays of direct application of fog and at different exposure times (1, 2, 4, 8, 16 and 32 min). **B** Front view of the position of stainless steel coupons in the second assay. Counterfog scope is studied for different radial distances (5, 10 and 15 cm) and at different times (2, 8, 16 and 32 min)

The dried contaminated test coupons were placed vertically in the disinfection area. All decontamination runs were performed in a closed room, projecting the cone at a distance of 2 m [from the nozzle (point of action) to the impact area where the coupons were located (point of application)] by means of a horizontal sweep at 0.4 m/s (Fig. 1). Environmental parameters were measured but not controlled during the surface disinfection experiments. At the moment of these experiments there was a temperature of 14 °C and 70% of relative humidity.

Two kinds of assays were done, one regarding sodium hypochlorite (NaClO) concentrations and the other one to study the reach in the radial distance of COUNTERFOG technology.

For the first set of assays three different concentrations of NaClO were used: 0.7, 1.2 and 1.8%. Sodium hypochlorite was diluted in pure water for the preparation of these concentrations. Following the fog application with the corresponding concentration, test coupons (20 × 9 mm) were collected at different times (1, 2, 4, 8, 16 and 32 min), placed in an eppendorf tube containing 990 µl of 1 × phage diluent and kept at 4 °C until sample processing (Fig. 1A).

For the second assay, the coupons were placed at 3 different radial distances from the point of application (5, 10 and 15 cm) to assess the scope of disinfection of the COUNTERFOG nozzle. Following the fog application with 1.2% NaClO, test coupons were collected at different times (2, 8, 16 and 32 min), placed in an eppendorf tube containing 990 µl of 1 × phage diluent and kept at 4 °C until sample processing (Fig. 1B).

For all of the assays a control sample, not subjected to the disinfection process, was taken.

### Sample Processing and Quantification of Viral Titer

For each sample serial dilutions (1/10) were made to allow the counting of the phage plaques. Each of them was tittered in duplicate following the two- step double- agar overlay method mentioned before. When a suspension of an infective phage, in this case ɸ29, is spread over the lawn of susceptible bacterial cells (*Bacillus subtillis*)*,* the phage attaches the bacterial cell, replicate inside it, and kills it during its lytic release (Tankeshwar, 2018). The lysis plaques can be seen over the bacterial lawn.

The original solution of ɸ29 phage was also plated to check the viral titre in the initial solution and a negative control with no virus was sampled in one of the wells. For these assays, the plating was done in 6-well plates instead of petri dishes due to the high number of samples. The plates were incubated overnight at 37 °C (approximately 24 h). After incubation, plaque-forming units (PFU) were counted manually.

### Disinfection Efficacy Calculations

Efficacy calculations were done following the instructions by Rogers et al. (2005). The log10 reduction was calculated using the following equation:

1 $${\text{Log}}\,{\text{Reduction}}\, = \,{\text{Log}}_{10} \left( \frac{N0}{N} \right)$$

where *N*0 is the mean number of viable organisms recovered from the control coupons (negative control), and *N* is the mean number of viable organisms recovered from coupons after decontamination.

Reduction percentage was calculated for each of the samples by comparing the number of PFU/ml recovered from control coupons with the number of PFU/ml recovered from sample coupons, according with the following equation:

2 $$P = 100 \times \frac{N0 - N}{{N0}}$$

All viral titer (PFU/ml) calculations, averaging and determination of standard deviations were performed in Microsoft Excel 2007.

## Results

### Effect of Sodium Hypochlorite Concentration on Disinfection

Three decontamination runs using COUNTERFOG® technology were conducted. For each one, a different concentration of NaClO was used: 0.7, 1.2 and 1.8%, respectively. For each of the runs a control sample, not subjected to decontamination, was taken. The samples were subjected to a unique application with COUNTERFOG® SDR-F05A+ equipment.

Exposure of test coupons contaminated with ɸ29 phages to sodium hypochlorite fog resulted in a reduction of viable virus that varied according to the concentration used and the exposure time. According to the UNE-EN 14476:2014 + A2 and UNE-EN 16777:2019 standards, an assay is valid if the titer of the test suspension is high enough to allow a decimal logarithmic reduction of the titer of at least 4 (AENOR, 2019, 2020). This value (4-log reduction or higher in virus recovery) allows to determine an effective disinfection procedure (Krug et al., 2012).

For a concentration of 0.7% NaClO, 2 min of exposure to this disinfectant was enough for a Log_10_ reduction of 3.50 and of > 6.50 in 16 min (Table 1). For higher NaClO concentrations (1.2% and 1.8%) there was a visible reduction of > 6.50 logs of viable ɸ29 phage within 2 min of exposure time. The average decontamination efficacy was > 6 log reduction for both sodium hypochlorite concentrations (Tables 2, 3).

**Table 1:** Disinfection efficacy results of bacteriophage ɸ29 following 0.7% NaClO fog created with COUNTERFOG®

Exposure time (min)	PFU/ml (Mean ± SD)	Log_10_ reduction
Control (non-treated)	1.58 ± 0.10 × 10^8^	NA
1	5.25 ± 0.28 × 10^6^	1.48 ± 0.02
2	4.53 ± 0.53 × 10^4^	3.54 ± 0.05
4	< 50^a^	> 6.50
8	4.80 ± 0.42 × 10^3^	4.52 ± 0.04
16	< 50^a^	> 6.50
32	< 50^a^	> 6.50

Values are expressed as mean ± SD from duplicates of each test sample

*NA* not applicable

^a^Lower limit of virus detection

**Table 2:** Disinfection efficacy results of bacteriophage ɸ29 following 1.2% NaClO fog created with COUNTERFOG®

Exposure time (min)	PFU/ml (Mean ± SD)	Log_10_ reduction
Control (non-treated)	1.60 ± 0.21 × 10^8^	NA
1	< 50^a^	> 6.51
2	75 ± 106	6.33 ± 4.26
4	< 50^a^	> 6.51
8	< 50^a^	> 6.51
16	< 50^a^	> 6.51
32	< 50^a^	> 6.51

Values are expressed as mean ± SD from duplicates of each test sample

*NA* not applicable

^a^Lower limit of virus detection

**Table 3:** Disinfection efficacy results of bacteriophage ɸ29 following 1.8% NaClO fog created with COUNTERFOG®

Exposure time (min)	PFU/ml (Mean ± SD)	Log_10_ reduction
Control (non-treated)	1.73 ± 0.18 × 10^8^	NA
1	4.95 ± 0.14 × 10^3^	4.54 ± 0.01
2	< 50^a^	> 6.54
4	< 50^a^	> 6.54
8	< 50^a^	> 6.54
16	< 50^a^	> 6.54
32	< 50^a^	> 6.54

Values are expressed as mean ± SD from duplicates of each test sample

*NA* not applicable

^a^Lower limit of virus detection

### Range of Action of COUNTERFOG®

In another run of disinfection, stainless steel coupons were placed at different radial distances from the point of application of Counterfog to study the disinfection reach of the fog application. The test coupons were placed at 5, 10 and 15 cm from the point of application and they were collected at different exposure times. The disinfectant concentration used was 1.2% NaClO.

The reduction percentage was also calculated for this assay (Table 4). At a radial distance of 5 cm from the point of fog application we obtained a 5.48 log reduction at 8 min (99.9997% reduction). At 10 cm it took 32 min to obtain a Log_10_ reduction of 5.34. And regarding the results obtained at a radial distance of 15 cm from the point of application the reduction range was 74–98%. At all exposure times there was approximately 1 log of reduction.

**Table 4:** Disinfection efficacy results of bacteriophage ɸ29 following 1.2% NaClO fog created with COUNTERFOG® at different radial distances from the area of fog application

Exposure time (min)	PFU/ml (Mean ± SD)	Log_10_ reduction	Reduction (%)
5 cm			
Control (non-treated)	1.60 ± 0.21 × 10^8^	NA	NA
2	5.03 ± 0.25 × 10^5^	2.50 ± 0.02	99.68
8	5.25 ± 1.77 × 10^2^	5.48 ± 0.15	99.9997
16	< 50^a^	> 6.51	99.99997
32	< 50^a^	> 6.51	99.99997
10 cm			
Control (non-treated)	1.60 ± 0.21 × 10^8^	NA	NA
2	7.43 ± 0.39 × 10^6^	1.33 ± 0.02	95.36
8	2.78 ± 0.10 × 10^6^	1.76 ± 0.02	98.26
16	2.40 ± 0.07 × 10^5^	2.82 ± 0.01	99.85
32	7.25 ± 2.47 × 10^2^	5.34 ± 0.15	99.9995
15 cm			
Control (non-treated)	1.60 ± 0.21 × 10^8^	NA	NA
2	1.58 ± 0.60 × 10^7^	1.01 ± 0.17	90.13
8	3.38 ± 0.74 × 10^6^	1.68 ± 0.10	97.89
16	1.45 ± 0.45 × 10^7^	1.04 ± 0.15	90.94
32	4.23 ± 0.60 × 10^7^	0.58 ± 0.06	73.56

Values are expressed as mean ± SD from duplicates of each test sample

*NA* not applicable

^a^Lower limit of virus detection

**Fig. 2:**
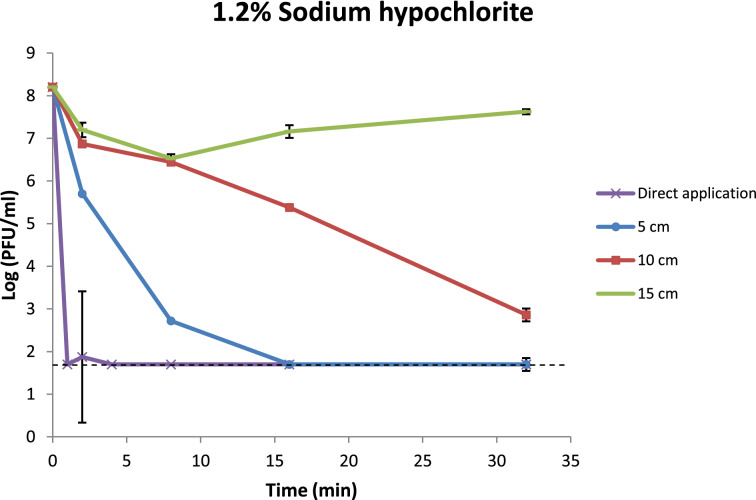
Behavior of bacteriophage φ29 when exposed to 1.2% NaClO. Each curve represents the virus behavior at different radial distances from the area of application (direct application, 5 cm, 10 cm and 15 cm). Samples were subjected to disinfection for different exposures times (1, 2, 4, 8, 16 and 32 min for direct application; and 2, 8, 16, 38 min for the radial distances). The error bars represent the standard deviation of duplicates for each sample. Dashed line indicates the lower limit of virus detection

The behavior of ɸ29 phage when exposed to 1.2% NaClO is shown in Fig. 2 along with the comparison of the viral inactivation curves at different radial distances from the point of application (Fig. 2). As it is expected viral inactivation decreases with the distance as the NaClO fog has a reach limit. Counterfog disinfection produces a fast drop of virus survival when the application is direct and at a radial distance of 5 cm. The inactivation curves show that at 10 cm it takes 4 times longer to reach the same inactivation than at a distance of 5 cm. At 15 cm there is not meaningful disinfection.

## Discussion

In this paper we present the ability of COUNTERFOG® to decontaminate stainless steel surfaces contaminated with bacteriophage ɸ29. We used bacteriophage ɸ29 mainly due to its safe and easy use. It is comparable to SARS-CoV-2 in terms of size, being one of the smallest bacteriophages of *Bacillus subtilis* (Anderson et al., 1966). In addition, phage ɸ29 is very accessible and has been extensively studied as a model for viral replication (Li et al., 2020). We think bacteriophage ɸ29 may be a good virus model for studying disinfection procedures. Non-enveloped viruses (as it is bacteriophage ɸ29) are generally more resistant to disinfectants than enveloped viruses (such as SARS-CoV-2 or hepatitis B and C viruses) (Lin et al., 2020; Twomey et al., 2003). For this reason, demonstrating the efficacy of any disinfection procedure with this virus makes it applicable to a wide range of other less resistant viruses.

COUNTERFOG® technology has been recently developed for the rapid disinfection of air and surfaces. The results obtained in this study showed that this system is capable of efficiently disinfecting stainless steel surfaces. Stainless steel is a common surface for study of viral stability, and has been used to study the persistence on a number of viruses such as Ebola virus, hepatitis virus, Influenza A and Coronaviruses (Riddell et al., 2020).

The physical principle that governs the operation of COUNTERFOG® technology is the droplet and micro–nano-CBRN particles dynamics. When these substances are floating in air, they are absolutely dragged by airflow. The smaller they are, the longer they remain suspended in air and it is more difficult to counteract them. This implies that they can only collapse or coalesce with droplets of liquid of a similar size (Pérez- Díaz et al., 2021). The fog is projected to the floor or other surfaces, and in the case of infectious agents, it’s essential to disinfect these surfaces. COUNTERFOG® allows the addition of any type of disinfectant; this is important because the nanometric droplets will not only capture the agent but also neutralized it. Furthermore, it is known that inanimate surfaces are a potential route of virus transmission, if microdroplets expelled when talking or coughing settle on them (Fedorenko et al., 2020).

Diluted sodium hypochlorite was applied with COUNTERFOG® system. After only 2 min of Counterfog application with 0.7% sodium hypochlorite there was a log reduction of 3.50, being a 4-log reduction the measure established for an efficient disinfection (AENOR, 2019, 2020). After 16 min of exposure a log reduction of > 6.50 is obtained. Increasing NaClO concentration we obtained the same log reduction in less time, (2–4 min; 6.50 log reduction). This means that a concentration of 1.2% is enough for obtaining almost 100% of surface reduction in a very short time, which means a very efficient surface disinfection. Therefore, we can observe that the efficiency of the disinfection depends on the concentration of the disinfectant and the exposure time to this disinfectant, as it is demonstrated by the Chick–Watson law for disinfection kinetics.

The experiments aiming to study the range of action of COUNTERFOG® and the radial distance to which the action of this system is effective showed that at 5 cm from the point of application a 5.5-log reduction was seen within 8 min of exposure. However, to be able to observe an efficient disinfection at a radial distance of 10 cm it was necessary an exposure time of 32 min. This gives us an insight of what is the range of action of the COUNTERFOG® nozzle but the purpose of this technology is to get an efficient disinfection in the shortest period of time possible. At a radial distance of 15 cm there was no effective reduction of the virus. These results imply that Counterfog has an effective range of action within a radius of 5 cm.

The results obtained in this study complement and extend the ones obtained in Pérez- Díaz et al., 2021. In this study, some first assays were performed using different microorganisms. Similarly, stainless steel surfaces were contaminated with several bacterial strains and disinfected using COUNTERFOG® technology. The results showed an average reduction of 3 orders of magnitude for the different pathogenic microorganisms assessed (Pérez- Díaz et al., 2021). Preliminary tests were also done with bacteriophage ɸ29; however, a limitation in this study is that the initial bacteriophage ɸ29 titer was a little bit low to clearly detect the efficiency in the inactivation. In addition, the present study contributes determining the optimal NaOCl concentration and exposure time for effective disinfection.

Fogging decontamination has several advantages over the other decontamination methods as mentioned above (Rogers et al., 2009; Wood et al., 2013). Specifically, COUNTERFOG® ejects a cone of pressurized fog that quickly fills a room, so it requires less time to decontaminate. Although the inactivation rate depends on the disinfectant, its concentration and the exposure time; Counterfog allows the application time of this disinfectant to be reduced. For example, for an application velocity of 0.4 m/s it takes 11 min of application for the surface disinfection of a 12 m^2^ room. The results in this study demonstrated that a single pass of Counterfog is enough for an appreciable disinfection. A lot of standards (AENOR) and regulations are applied for biocides (bactericides, virucides…); however, these standards do not refer to the mode of application. Counterfog diminishes the time needed for application of the disinfectants thanks to its nanometric droplets’ principle.

Some other advantages are its ability to reach a distance of 2 m, making it a perfect technology for large-scale decontamination; its ability to create fogs of any type of disinfectant, significantly saving in the use of the biocide and the generation of minimal liquid waste and consequent damage to the environment.

There may be some possible limitations in this study. The low number of conducted replicas supposed some limitations when performing statistical calculations. The results, however, are very conclusive with respect to the disinfection efficiency since there is a reduction of > 6.50 logs. Furthermore, these results are consistent with results obtained in previous unpublished assays regarding rate of disinfection. On the other hand, the temperature and relative humidity conditions were those established by the place and time of the test, not being controlled variables. In this study only the disinfection efficiency has been assessed, an interesting approach would be the comparison, under the same settings, to other relevant disinfection technologies.

For future research it would be interesting to compare the disinfection efficiency in different types of material with porous and non-porous characteristics that are used in common buildings and areas. The use of different disinfectants can also be assessed, in order to find an effective disinfectant that causes no damage to health and to the environment. In addition, other types of viruses as RNA virus models can also be tested to determine the efficacy of this technology in viruses with other characteristics. The COVID19 pandemic has clearly revealed the need of a system capable of removing infectious agents from the air. An important approach that should be conducted is the air sanitation of viral particles with COUNTERFOG® technology.

## Conclusion

In summary, the aim of this study was to assess the efficiency of COUNTERFOG® technology in disinfecting viral contaminated surfaces. Surface disinfection efficacy depends on the concentration and exposure time of the disinfectant. A concentration between 0.7 and 1.2% sodium hypochlorite is enough for an efficient ɸ29 phage reduction in less than 5 min of exposure time due to the thin layer of disinfectant that cover the surface when the fog is applied. COUNTERFOG® system is highly effective in a radius of 5 cm, making the disinfection of the place a fast process.

More research and assays are needed for the improvement of this technology, but the results presented in this study are a small demonstration of the disinfecting capability of COUNTERFOG® with a bacteriophage model. For this reason, this technology can be used as a tool for elimination and prevention of infectious diseases, especially respiratory diseases which are easily transmitted through the environment.

## Data Availability

All data generated or analysed during this study are included in this published article.
